# Time-to-event analyses of lower-limb venous thromboembolism in aged patients undergoing lumbar spine surgery: a retrospective study of 1620 patients

**DOI:** 10.18632/aging.102364

**Published:** 2019-10-15

**Authors:** Liang Li, Zhaohui Li, Yachong Huo, Dalong Yang, Wenyuan Ding, Sidong Yang

**Affiliations:** 1 Department of Spine Surgery, The Third Hospital of Hebei Medical University, Shijiazhuang 050051, PR China; 2 Hebei Provincial Key Laboratory of Orthopaedic Biomechanics, Shijiazhuang 050051, PR China

**Keywords:** VTE, DVT, thrombosis, spine surgery, risk factor

## Abstract

After spine surgery, venous thromboembolism (VTE) is not uncommon in aged patients. This study investigates time-to-event risk factors of postoperative VTE based on medical records of aged patients (age≥60 yr) between January 2013 and December 2018. All participants had undergone lower extremity ultrasonography pre- and postoperatively at the first, second, fourth, eighth, and twelfth weeks. Mann-Whitney U tests and chi-square tests were used for univariate analyses, and Cox regression was utilized for multivariate analyses. A total of 1620 cases were recruited, VTE group (N=382, 23.6%) and non-VTE group (N=1238, 76.4%), aged 67 (IQR 11) years and 65 (IQR 6) years, respectively. The univariate analyses indicated significant differences between the VTE and non-VTE groups regarding advanced age, VTE history, hypertension, fusion, hospital stay, FIB, HDL, D-dimer, and TC (all P<0.05). The Cox regression showed that advanced age (OR=1.108; 95% CI, 1.091–1.126), VTE history (OR=4.962; 95% CI, 3.849–6.397), and hypertension (OR=1.344; 95% CI, 1.084–1.667) were the risk factors for postoperative VTE (P<0.05). The time-to-event analyses indicated that the hazard of VTE formation was highest in the first postoperative week. In conclusion, advanced age, VTE history, and hypertension are main risks for VTE formation, particularly in the first postoperative week.

## INTRODUCTION

It is well-known that venous thromboembolism (VTE) is a major postoperative complication and a common and potentially lethal disease; VTE includes pulmonary embolism (PE) and deep venous thrombosis (DVT) [1–10]. VTE may cause severe morbidity, lead to poor life quality, and even death because of PE. It is estimated that around 50% of untreated DVT events can be complicated by PE; likewise, if untreated, 50% to 80% of PE cases are related to DVT events [5, 11]. Previous studies have uncovered that VTE events are possibly associated with the following risk factors: D-dimer, trauma, smoking, obesity, advanced age, neurological deficit, immobilization, malignancy, blood transfusion, major surgery, hospitalization, and inherited hypercoagulable state [12–16].

The risk for a VTE event increases after patients undergo surgical operations, such as a spinal operation [17, 18]. Annually, worldwide spinal operations are on the rise [19, 20]. Hence, it is important to seek proper management to reduce possible VTE events among patients who undergo spinal operations. In terms of patients undergoing spinal surgeries, the risks of VTE formation probably include a loss of muscular tension, postoperative bed rest, long-time horizontal abdominal position, and vascular compression by intraoperative retractors. It has been well documented in our previous study that around 15% of patients who undergo spinal operations—those without appropriate prophylaxis—progress to experiencing DVT events [21]. Particularly, a VTE event is considered to be highly associated with advanced age; accordingly, in previous studies, advanced age has been identified as an important risk contributing to VTE events after spine surgeries [5, 6, 22, 23].

Although the various risk factors of VTE events after spinal surgeries have been reported in previous studies [7, 24], understanding these findings is still elusive because of the selection of patient samples; this is likely to create more difficulties in understanding VTE development after spine surgery. Furthermore, the time to VTE events for aged patients after undergoing spinal operations has yet to be well elucidated. Therefore, the current study aims to uncover the occurrence and timing of VTE events after spinal operations by only recruiting an aged population (age≥60 yr).

## RESULTS

### Baseline data

The demographic data are shown in Table 1. In total, 1,620 cases were identified and included in the current study. All patients had undergone lower-limb ultrasonography checks pre- and postoperatively at the first, second, fourth, eighth, and twelfth weeks. All cases were automatically sorted into the following two groups according to their VTE status: the VTE group (N=382, 23.6%) and non-VTE group (N=1238, 76.4%). All VTE events were asymptomatic, and most were calf DVT events (N=356, 93.2%). Specifically, the VTE group included 194 males and 188 females, and the non-VTE group included 668 males and 570 females. There is no statistical significance between the VTE group and non-VTE group in terms of gender (P=0.227). In addition, there are no differences regarding regional distribution, diabetes, and heart disease (all P>0.05).

**Table 1:** Baseline demographic data.

**Items**	**VTE (N=382)**	**non-VTE (N=1238)**	**-value**	***p*-value**
Age	67 (IQR 11) yrs	65 (IQR 6) yrs	--	*< 0.001*
Sex			1.180	0.277
Male	194	668		
Female	188	570		
Region			0.074	0.785
Rural	261	855		
Urban	121	383		
VTE history			135.1	*< 0.001*
Yes	79	38		
No	303	1200		
Hypertension			15.47	*< 0.001*
Yes	122	273		
No	260	965		
Diabetes mellitus			3.620	0.057
Yes	44	103		
No	338	1135		
Heart disease			0.181	0.671
Yes	17	49		
No	365	1189		
Fusion			4.649	*0.031*
Yes	349	1169		
No	33	69		
Hospital stay	15 (IQR 6) days	14 (IQR 5) days	--	*0.002*

VTE, venous thromboembolism; Age and hospital stay are presented as median and interquartile range (IQR).

The median age in the VTE group was 67 (IQR 11) years, while it was 65 (IQR 6) years in the non-VTE group. The Mann-Whitney U test showed that the participants in the VTE group were older compared with the non-VTE group (P<0.001). Compared with the non-VTE group, more patients in the VTE group had a previous VTE history and were suffering from hypertension (both P<0.001), but fewer patients in the VTE group underwent fusion surgery (P=0.031). The median length of stay for the VTE patients was 15 days (IQR 6), which is slightly longer than the non-VTE group (14 days, IQR=5; P=0.002).

### Univariate analyses associated with postoperative VTE

As shown in Table 2, the surgically related data included the duration of surgery, blood loss, blood transfusion, and the length of incision, all of which were retrieved from the patient operation notes. As a result, all comparisons of surgical data between the VTE group and the non-VTE group turned out to be statistically insignificant (all P>0.05). In addition, comparisons regarding FIB, D-dimer, HDL, and TC showed statistical significance between the two groups (all P<0.05). However, the comparisons of other biochemical items, including PTA, TT, LDL, T-BIL, D-BIL, and I-BIL, showed no significant difference (all P>0.05). Moreover, the BMI data were compared between the VTE group and non-VTE group, with no difference being identified (P=0.226).

**Table 2:** Univariate analyses associated with postoperative VTE.

**Items**	**VTE (N=382)**		**Non-VTE (N=1238)**	**Mann-Whitney U test**	
	**Median(IQR)**		**Median(IQR)**	**Z-value**	***p*-value**
Surgical duration	135 (75) min		140 (75) min	-1.124	0.261
Blood loss	400 (500) ml		400 (400) ml	-0.040	0.968
Blood transfusion	0 (400) ml		0 (126) ml	-1.676	0.094
Incision length	14 (8.25) cm		14 (8.00) cm	-0.145	0.885
PTA	109.4 (20)%		109.6 (20)%	-1.099	0.272
FIB	2.92 (0.71) g/L		2.78 (0.83) g/L	-2.576	*0.010*
TT	14.6(1.6) s		14.5 (1.7) s	-0.272	0.786
D-dimer	0.14 (0.15) mg/L		0.11 (0.10) mg/L	-5.958	*<0.001*
HDL	1.145 (0.40) mmol/L		1.11 (0.35) mmol/L	-3.301	*0.001*
LDL	3.12 (1.10) mmol/L		3.04 (1.12) mmol/L	-1.733	0.083
TC	4.76 (1.45) mmol/L		4.63 (1.30) mmol/L	-1.992	*0.046*
T-BIL	12.85 (5.70) umol/L		12.50 (5.73) umol/L	-1.083	0.279
D-BIL	4.00 (2.20) umol/L		3.90 (2.10) umol/L	-0.700	0.484
I-BIL	8.7 (4.43) umol/L		8.30 (4.50) umol/L	-1.545	0.122
BMI	25.33 (4.01) kg/m^2^		24.77 (4.40) kg/m^2^	-1.211	0.226

VTE, venous thromboembolism; IQR, interquartile range; PTA, prothrombin time activity; FIB, fibrinogen; TT, thrombin time; HDL, high density lipoprotein; LDL, low density lipoprotein; TC, total cholesterol; T-BIL, total bilirubin; D-BIL, direct bilirubin; I-BIL, indirect bilirubin; BMI, body mass index.

### Time-to-event analysis of VTE

**Figure 1:**
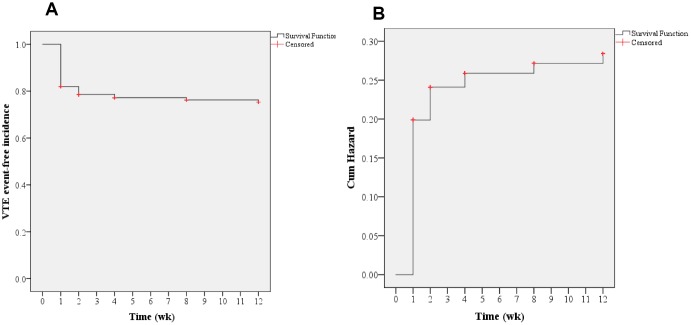
**Overall Kaplan-Meier analysis.** (**A**) Time-to-event analysis of postoperative VTE incidences; (**B**) Cumulative hazard model (log-rank test, P<0.001).

In the current study, all included patients had undergone lower extremity ultrasonography pre- and postoperatively at the first, second, fourth, eighth, and twelfth weeks. Thus, time-to-event Kaplan-Meier survival analyses were carried out to determine the potential risks causing postoperative VTE. As shown in Figure 1, an overall survival curve indicates that most VTE events occurred in the first week after spine surgery, followed by the second week (P<0.001), which was further supported by a cumulative hazard model.

### Cox regression analysis

**Figure 2:**
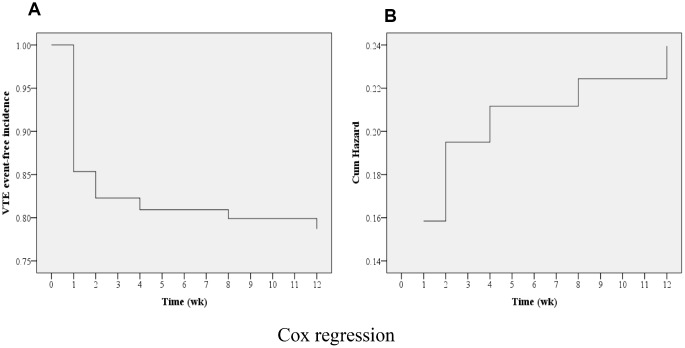
**Cox regression multivariable analysis.** (**A**) Time-to-event analysis of postoperative VTE incidences; (**B**) Cumulative hazard model (P<0.001).

**Figure 3:**
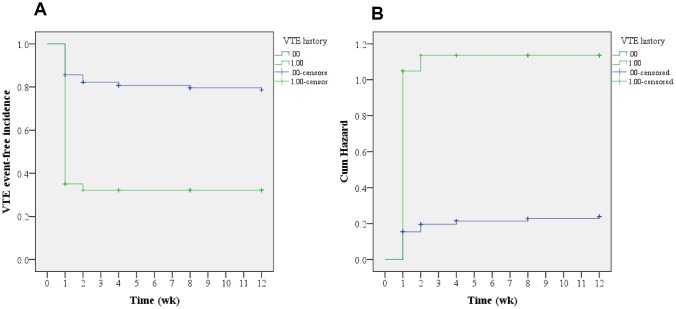
**Kaplan-Meier analysis regarding VTE history.** (**A**) Time-to-event analysis of postoperative VTE incidences related to VTE history; (**B**) Cumulative hazard model (log-rank test, P<0.001).

**Figure 4:**
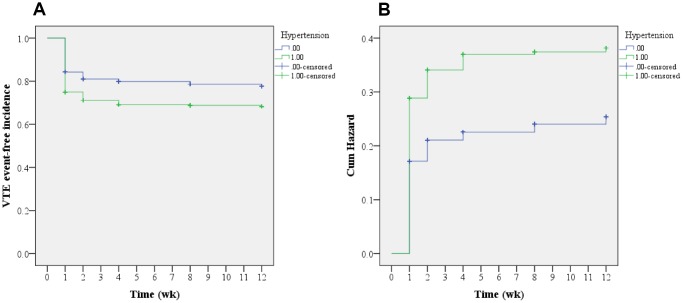
**Kaplan-Meier analysis regarding hypertension.** (**A**) Time-to-event analysis of postoperative VTE incidence related to hypertension; (**B**) Cumulative hazard model (log-rank test, P<0.001).

As shown in Table 3, a Cox regression analysis was carried out to further locate the risks of postoperative VTE. Consequently, advanced age (OR=1.108; 95% CI, 1.091–1.126), VTE history (OR=4.962; 95% CI, 3.849–6.397), and hypertension (OR=1.344; 95% CI, 1.084–1.667) were identified as the risk factors of postoperative VTE formation (all P<0.05). As shown in Figure 2, the cumulative survival curve indicated that most VTE events occurred in the first week after spine surgery (P<0.001), which was further confirmed by a cumulative hazard model (P<0.001). Furthermore, time-to-event Kaplan-Meier survival analyses were performed to determine the influence on postoperative VTE regarding VTE history and hypertension. Clearly, both VTE history and hypertension increased the risk of postoperative VTE formation, which is shown, respectively, in Figures 3, 4 (log-rank tests, both P<0.001). It revealed that the hazard growth mostly occurred in the first week after spine surgery, regardless of VTE history and hypertension (log-rank tests, both P<0.001).

**Table 3:** Cox regression analysis of postoperative VTE.

**NO.**	**Items**	**B**	***p*-value**	**Exp(B)**	**95% CI for Exp(B)**
X1	Sex	0.043	0.709	1.044	(0.832, 1.311)
X2	Age	0.103	*<0.001*	1.108	(1.091, 1.126)
X3	Region	-0.019	0.862	0.981	(0.787, 1.221)
X4	Hospital stay	0.010	0.343	1.010	(0.989, 1.032)
X5	VTE history	1.602	*<0.001*	4.962	(3.849, 6.397)
X6	Hypertension	0.296	*0.007*	1.344	(1.084, 1.667)
X7	Diabetes	0.163	0.337	1.177	(0.844, 1.642)
X8	Heart disease	-0.337	0.190	0.731	(0.431, 1.182)
X9	Fusion	-0.313	0.166	0.731	(0.470, 1.139)
X10	Levels of fusion	0.102	0.232	1.107	(0.937, 1.309)
X11	Surgical duration	-0.001	0.311	0.999	(0.996, 1.001)
X12	Blood loss	<0.001	0.514	1.000	(0.999, 1.000)
X13	Blood transfusion	<0.001	0.237	1.000	(1.000, 1.001)
X14	Incision length	0.002	0.871	1.002	(0.981, 1.023)
X15	PTA	0.006	0.123	1.006	(0.998, 1.013)
X16	FIB	<0.001	0.997	1.000	(0.843, 1.186)
X17	TT	0.021	0.673	1.021	(0.927, 1.125)
X18	D-dimer	0.095	0.464	1.099	(0.853, 1.417)
X19	HDL	0.113	0.148	1.119	(0.961, 1.305)
X20	LDL	0.082	0.352	1.086	(0.913, 1.290)
X21	TC	0.007	0.933	1.007	(0.862, 1.176)
X22	TBIL	-0.002	0.955	0.998	(0.926, 1.075)
X23	DBIL	0.042	0.470	1.043	(0.930, 1.169)
X24	IBIL	-0.004	0.921	0.996	(0.924, 1.074)
X25	BMI	0.004	0.826	1.004	(0.972, 1.037)

VTE, venous thromboembolism; CI, confidence interval; PTA, prothrombin time activity; FIB, fibrinogen; TT, thrombin time; HDL, high density lipoprotein; LDL, low density lipoprotein; TC, total cholesterol; T-BIL, total bilirubin; D-BIL, direct bilirubin; I-BIL, indirect bilirubin; BMI, body mass index.

## DISCUSSION

To the best of our knowledge, VTE is not uncommon in aged patients after lumbar spine surgery, regardless of fusion surgery; VTE events are reported to be highly correlated with the aging population [23]. However, the time-to-event analysis in aged patients has not been well documented. In our hospital, according to follow-up procedures, all patients who have undergone posterior spinal surgeries (regardless of fusion) are routinely requested to return for lower extremity ultrasonography pre- and postoperatively at the first, second, fourth, eighth, and twelfth weeks during their treatment. The current study focused on the time-to-event risk factors of postoperative VTE by utilizing medical records of aged 60 and over patients. Overall, a total of 1,620 cases were identified and recruited for the data analyses. Thus, the sample size is large enough to generate reliable results. As a result, 382 (23.6%) VTE events were found. All VTE events were asymptomatic, and most were calf DVT events (N=356). Univariable analyses did not indicate any statistical significances between the VTE group and non-VTE group in terms of gender proportion, regional distribution, diabetes, and heart disease.

Other studies have investigated VTE incidence after spine surgeries and reported a variety of data [7, 25–28]. Agarwal et al. [25] reported that only 0.6% of patients were documented with VTE events, including 28 DVT and 42 PE. This might be because the authors did not generate the results based on regular follow-ups using ultrasonography as we have done in the current study. Recently, Inoue et al. [26] performed an investigation of VTE events using indirect multidetector computed tomography in a prospective study including 105 cases that underwent spinal surgeries; they found that all VTE events were asymptomatic. The preoperative incidence of asymptomatic VTE was 4.8%, and the postoperative was 13.0%; the incidence of asymptomatic PE was 2.9% preoperatively and 8.0% postoperatively; and the incidence of asymptomatic DVT was 3.8% preoperatively and 6.0% postoperatively. The age, gender, BMI, surgical duration, blood loss, and specific comorbidities, including heart disease, hypertension, diabetes, previous VTE, and previous anticoagulant intake, were excluded from the risk factors. To a certain extent, the results in our study are in line with the reports above. Associated with postoperative VTE, our univariate analyses indicated that the duration of surgery, blood loss, and transfusion and the length of an incision between the VTE group and non-VTE group turned out to be statistically insignificant (all P>0.05). However, our work identified that advanced age, hypertension, VTE history, and prolonged hospital stay were risk factors for VTE formation. Compared with the non-VTE group, more patients in the VTE group had a previous VTE history and suffered from concurrent hypertension, but fewer patients in the VTE group underwent fusion surgery, which was more likely to be a protective factor. What may be the cause of such differences? As we can see, the reading of these findings is still somewhat questionable because of the selection of patient samples; this is likely to make the understanding of VTE progression after spine surgery less clear.

In the current study, time-to-event Kaplan-Meier survival analyses were conducted to determine the potential risks of postoperative VTE events. Consequently, an overall survival curve indicated that most VTE events occurred in the first week after spine surgery, followed by the second week; this was further supported by a cumulative hazard model. In addition, Cox regression analyses were carried out for further identification of the potential risk factors of postoperative VTE. Consequently, advanced age (OR=1.108; 95% CI, 1.091–1.126), VTE history (OR=4.962; 95% CI, 3.849–6.397), and hypertension (OR=1.344; 95% CI, 1.084–1.667) were identified as the risk factors for postoperative VTE formation. Furthermore, time-to-event Kaplan-Meier survival analyses were performed to determine the influence on postoperative VTE regarding VTE history and hypertension. Clearly, both VTE history and hypertension increased the hazard of postoperative VTE formation. It revealed that the highest hazard incidences mostly occurred in the first week after spine surgery, regardless of VTE history and hypertension. Previously, McClendon et al. [27] performed a retrospective time-to-event analysis for VTE progression after spinal fusion ≥ five levels. Likewise, they also identified VTE history as an independent risk factor for VTE formation. Cloney et al. [8] reported that the cumulative incidence of VTEs rose linearly in the first two postoperative weeks and then plateaued. By contrast, our findings show that the likelihood of VTE formation is the highest in the first postoperative week, followed by the second postoperative week. The differences in these results may be because of the different participants included in the studies, with only aged patients being recruited in our study.

Taken together, the current study has detected some significant correlations that can help in better understanding VTE progression in aged patients who undergo spinal operations. In addition, the risk factors identified in the current study can be regarded as early alerts for postoperative VTE events. Indeed, our study suggests that an early mechanical and/or chemoprophylaxis should be applied to aged patients as soon as possible, particularly during the first week after spine surgery. As a guidance here, it would be helpful to reduce postoperative VTE incidence in vulnerable, aged patients. However, the retrospective nature of the current study is bound to induce some limitations; the most obvious one is the study’s dependence on the quality of the data documented in the participants’ medical records. In fact, as the largest spinal medical center in our region, the present study might have incorporated some patients suffering from more severe diseases. As such, a future study should be designed as a prospective, multicenter, randomized controlled trial with a large sample size; this future study should be able to reveal stronger evidence and identify more possible risk factors that contribute to VTE events after spine surgery in aged patients.

## CONCLUSIONS

In summary, the current study has identified that advanced age, VTE history, and hypertension are risk factors for postoperative VTE formation in aged patients, and VTE events are likely to occur mainly in the first postoperative week. Therefore, early mechanical and/or chemoprophylaxis should be applied to aged patients as soon as possible, particularly during the first week after spine surgery. As a guidance, it would be helpful to reduce postoperative VTE incidence in vulnerable, aged patients.

## PATIENTS AND METHODS

### Ethical statement

The present study was approved by the Ethics Committee of the Third Hospital of Hebei Medical University (Ethics Committee of Hebei Provincial Orthopedic Hospital). Informed consent was obtained in our department during the patient treatment, with their approval being given as participants in our studies, including the utilization of their medical records.

### Patients

In the present study, the medical records of aged participants (age≥60 yr) who had undergone spinal operations between January 2013 and December 2018 were collected in the spine surgery department. All patients had undergone lower-limb Doppler ultrasonography pre- and postoperatively at the first, second, fourth, eighth, and twelfth weeks.

In the current study, we retrospectively collected the patients’ medical records. The eligible patients identified in the present study should have complete records including the following medical information and data: the patient ID number; patient age; gender; length of stay; living region distribution; the body height and weight used to calculate body mass index (BMI); lower-limb ultrasonography for VTE; chronic disease history including diabetes mellitus, heart disease and hypertension; surgical procedures; fusion levels and the number; duration of surgery; blood loss; transfusion; the length of an incision; biochemical assays including thrombin time (TT); D-dimer; fibrinogen (FIB); prothrombin time activity (PTA); high-density lipoprotein (HDL); low-density lipoprotein (LDL); total bilirubin (T-BIL); direct bilirubin (D-BIL); indirect bilirubin (I-BIL); and total cholesterol (TC). All included participants should have undergone the same prophylaxis procedures of postoperative VTE, including physical mechanical procedures and chemoprophylaxis using low-molecular-weight heparin 5000 IU per day starting on the first postoperative day.

The exclusion criteria were the following: conservative treatment; percutaneous transforaminal endoscopic discectomy; and percutaneous kyphoplasty or vertebroplasty because such a patient would undergo a rapid hospital discharge after surgery. Also, patients with spinal cord injuries were excluded because of their much longer hospital stay. Additionally, a patient would not be included in the current study if he or she was suffering from VTE before spinal surgery or previously had taken anticoagulant drugs such as aspirin, warfarin, and clopidogrel in the week before admission into the hospital.

### Methods

The methods we used were conducted according to the approved guidelines. These investigators (LL, ZL, and YH) identified the eligible participants, collected complete medical records, and extracted the medical data. In addition, two other researchers (DY and SY) took responsibility for data preparation and set up the dataset for further statistical analysis. Additionally, three investigators (SY, LL, and ZL) performed the data analyses separately and finally discussed the results together. Any disagreements were solved by discussion among at least three authors.

### Statistical analyses

In the current study, SPSS software 18.0 (IBM SPSS Inc., Chicago, IL) was used for the data analysis. Statistical description of the data is shown with the median (interquartile range, IQR) because these data did not meet the criteria of normality and homogeneity of variance. Univariable analyses were performed using the Mann-Whitney U test and chi-square test (where applicable). Time-to-event analyses were performed using Kaplan-Meier survival analyses. A Cox regression model was utilized to investigate the potential risk factors of postoperative VTE formation in aged 60 and over patients undergoing spinal surgery. Significance was set as P<0.05, which was based on two-tailed tests.
